# A Scoping Review of 5‐HT_3_ Antagonist Antiemetic Medication Safety in the Management of Nausea and Vomiting of Pregnancy

**DOI:** 10.1002/pds.70449

**Published:** 2026-08-13

**Authors:** Shannon Morgan, Ebony Quintrell, Danielle J. Russell, Amina Rhaman, Aster Gebremedhin, Caitlin S. Wyrwoll, Erin Kelty

**Affiliations:** ^1^ Medical School, the University of Western Australia Perth Australia; ^2^ School of Population & Global Health, the University of Western Australia Perth Australia; ^3^ School of Human Sciences, the University of Western Australia Perth Australia

**Keywords:** 5‐HT_3_ receptor antagonist, antiemetics, granisetron, ondansetron, pregnancy

## Abstract

**Purpose:**

The prescribing of 5‐hydroxytryptamine 3 receptor and type 3 serotonin receptor (5‐HT_3_) antagonists, particularly ondansetron, for nausea and vomiting in pregnancy (NVP) has increased globally. However, evidence on the safety of these medications in pregnancy remains unclear. This review aims to examine the available literature surrounding the safety of 5‐HT_3_ antagonist medication use for NVP.

**Methods:**

Embase, Ovid Medline, Global Health, and CINAHL were searched from inception to 20 March 2024, updated on 7 May 2025. Full‐text, original articles available in English, examining the use of 5‐HT_3_ antagonist medications (ondansetron, granisetron, tropisetron, dolasetron, palonosetron, ramosetron) for NVP were included. Any maternal, neonatal, or child health outcomes were of interest.

**Results:**

Results were screened and extracted using Covidence and synthesized in Excel. The search strategy returned 1942 articles of which 39 unique, full‐text studies were included. Most papers examined ondansetron (34/39 papers, 87%) with limited evidence for granisetron (5/39 papers, 13%). Ondansetron use for NVP yielded minimal complications for maternal health and late pregnancy outcomes. No elevated risk of any congenital anomalies overall was observed. Although the risks of cardiac and orofacial anomalies were conflicting, the absolute changes in risk are minimal for either anomaly, especially considering potential confounding from study methodology and limited sample sizes.

**Conclusion:**

Ondansetron likely poses low risk of harm to maternal and neonatal health, especially considering the excess risks of untreated NVP; however additional research is required for certain congenital anomalies. Further investigation is also warranted into granisetron safety before its widespread use during pregnancy.

## Key Points

1. Use of 5‐HT3 antagonist medication (e.g., ondansetron, granisetron) is becoming more popular in pregnancy for nausea and vomiting.
2. Knowledge of their safety in pregnancy is insufficient and often conflicting.
3. Substantial amount of literature on ondansetron, suggests safe for pregnant people and neonates overall. Marginal risk of congenital anomalies should be weighed against risks of untreated nausea and vomiting.
4. Granisetron studies are limited, more data required before its widespread use.
5. Identifying true safety of these medications is complicated by heterogenous disease classification and limited sample sizes for detecting rare effects among current published literature.

## Plain Language Summary

Nausea and vomiting of pregnancy effects approximately 70% of pregnancies worldwide and can have devastating effects on the pregnancy if inadequately managed. Ondansetron and other medications with a similar mechanism of action (e.g., granisetron) are gaining popularity for treating nausea and vomiting in pregnancy despite unclear evidence of their safety. This review aims to examine the available literature surrounding the effects of these medications on the pregnant person and their child if used during pregnancy. Four health databases were searched for full‐text, original articles in English on this topic. Of the 1942 articles identified, 39 papers were included in this review. Most examined ondansetron (34/39 papers, 87%) with the remainder studying granisetron (5/39 papers, 13%). Ondansetron was of minimal harm to the pregnant person, however for the child, results were conflicting for the occurrence of birth defects of the heart and oral cavity. These are both rare anomalies and limitations in the existing studies may have influenced the reporting of these outcomes. Ultimately, ondansetron may be considered safe to use in pregnancy, especially when untreated nausea and vomiting can bring greater harm. More research is required for ondansetron and birth defects, and for granisetron before its widespread use.

## 1 Introduction

Nausea and vomiting of pregnancy (NVP) affects approximately 70% of pregnant people worldwide [1, 2]. NVP usually occurs between the fourth and tenth week of gestation and resolves by week 20 [3]. Severe NVP may persist throughout pregnancy accompanied by severe weight loss and acute starvation, which is referred to as hyperemesis gravidarum (HG) and affects 0.3%–3% of pregnancies [3, 4]. Both NVP and HG can significantly impact the wellbeing of the pregnant person [3, 4], as well as the physical and long‐term neurodevelopmental health of the fetus [1, 5, 6].

Recent management trends for NVP illustrate 5‐hydroxytryptamine 3 receptor and type 3 serotonin receptor (5‐HT_3_) antagonists have become a popular alternative for more severe cases of NVP [4, 7, 8]. This may be due to their efficacious and well‐tolerated use in non‐gravid populations [9]. These medications (e.g., ondansetron, granisetron) act by blocking serotonin action in the brain's chemoreceptor trigger zone and gastrointestinal vagal nerve terminals to elicit antiemetic effects [10]. Evidence for their safety in NVP is limited [4, 7, 8].

For ondansetron, recent meta‐analyses have identified a potential for teratogenicity of cardiac and orofacial anomalies [11, 12], as the critical periods for developing these malformations coincide with the typical period of ondansetron use for NVP [13]. Despite these concerns, its off‐label use for NVP has notably increased, from 4.3% of pregnancies in 2002 to 14% in 2014 in the United States (US) alone [14]. Similar trends have been observed in Australia and the United Kingdom (UK) [10, 15]. Thus, understanding the current literature on the safety of ondansetron, and 5‐HT_3_ antagonists more broadly, for the treatment of NVP is essential for ensuring the appropriate use of these medications.

## 2 Objective

This scoping review seeks to examine the extent of available evidence surrounding maternal, neonatal, and child safety with the use of 5‐HT_3_ antagonist medications for NVP.

## 3 Methods

The Preferred Reporting Items for Systematic Reviews and Meta‐Analyses Extension for Scoping Reviews (PRISMA‐ScR) checklist was used to guide the synthesis of this review. A study protocol was published on Open Science Framework on 16 March 2024 (https://doi.org/10.17605/OSF.IO/9M2SE).

### 3.1 Information Sources and Search Strategy

Embase, Ovid Medline, Global Health, and Cumulated Index to Nursing and Allied Health Literature (CINAHL) were searched from inception until 20 March 2024, and repeated on 7 May 2025. Search terms included the class of 5‐HT_3_ antagonist medications, with generic and branded names of specific medications. A broad filter validated by the Ovid Tools Expert Searches captured the pregnancy aspect of the search [16]. Exact search terms are presented in Table S1.

### 3.2 Eligibility Criteria

Primary research articles with exposure to any 5‐HT_3_ antagonist medication at any period during pregnancy were included. Comparisons to no treatment or alternative antiemetics (including complementary or alternative medicines) were included. Outcomes of interest were any changes in maternal, neonatal, or child health. Only published full‐texts available in English were examined.

We excluded all secondary research, review articles without original data, and case studies with fewer than ten exposed pregnancies. Studies using animal subjects or in vitro human models were also excluded. Studies using 5‐HT_3_ antagonists for conditions other than NVP were excluded (e.g., managing maternal side effects of spinal anesthesia for caesarean deliveries) [17, 18].

### 3.3 Study Selection and Data Extraction

Following the search, all articles were exported to Covidence for automatic deduplication. Title, abstract, and full‐text screening were performed independently by two members of the research team. Conflicts were resolved through group discussion. Relevant information from the full‐text articles was recorded using an electronic data extraction form designed for this review. This included data about the study setting, design, exposure classification, comparator group details, and health outcomes (maternal, neonatal, and/or child). Extraction was completed in duplicate by two authors independently, with the final data exported into an Excel spreadsheet for synthesis.

## 4 Results

### 4.1 Study Selection and Characteristics

**FIGURE 1:**
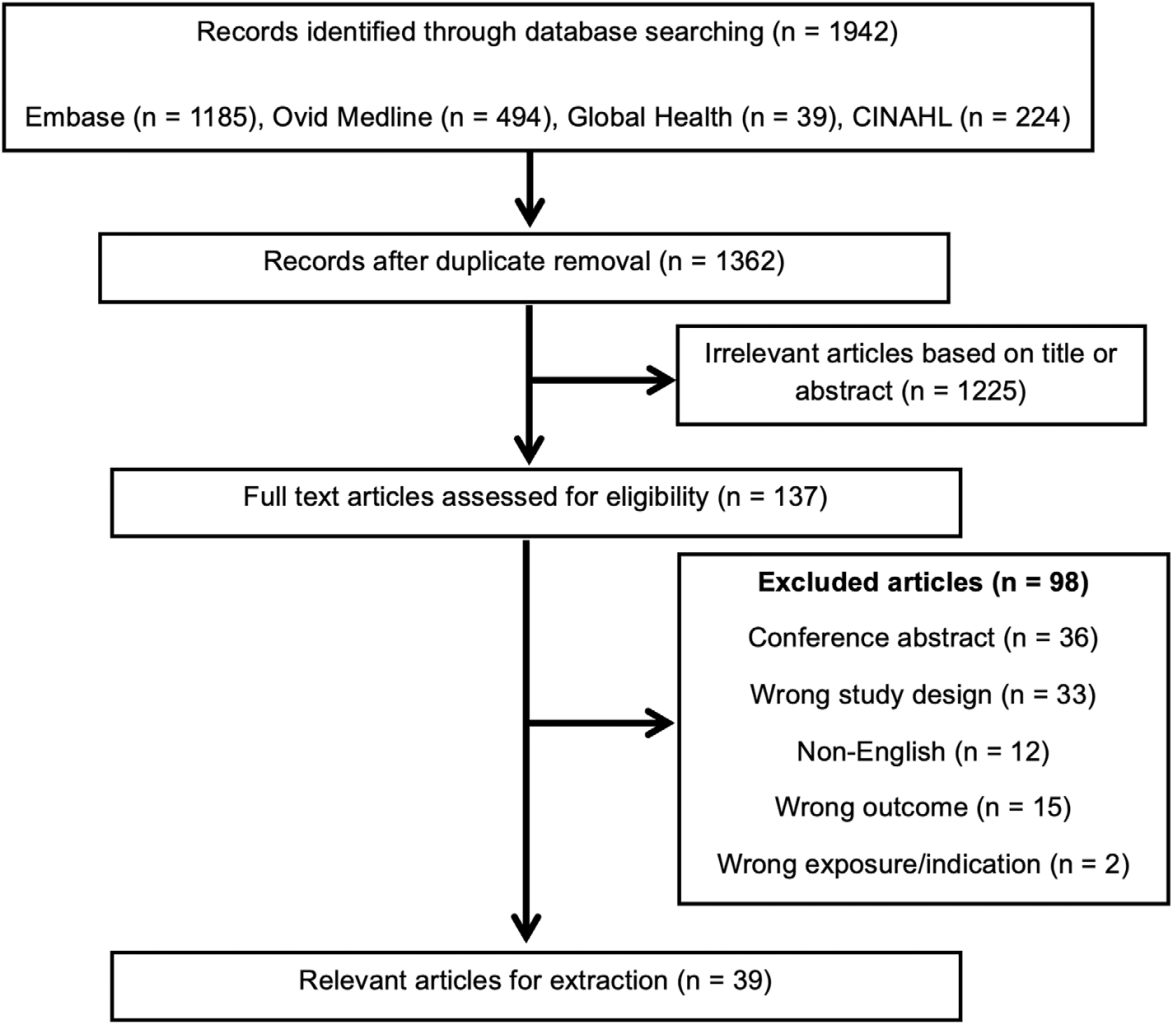
PRISMA flow diagram for the selection of included studies.

A total of 1942 articles were obtained, and 1362 entered screening after deduplication (Figure 1). Of the 137 full‐text papers deemed relevant, 39 articles were eligible for data extraction (Table 1). There were 20 cohort studies, nine randomised control trials (RCTs), seven case–control studies, two case series and a single pharmacokinetics study. Of the 39 studies, 20 (51%) were from the US, four (10%) from Canada and Iran each, three (8%) from Israel, and two (5%) from Sweden. Single studies originated from Australia, Denmark, Malaysia, Türkiye, India, and the UK. The majority focused on ondansetron (34 papers, 87%), with a handful considering granisetron (5 papers, 13%). Other 5‐HT_3_ antagonists were not examined. Werler et al. (2014) [50] broadly examined gestational medication exposure without specifying the condition requiring ondansetron. However, NVP is the only relevant indication for ondansetron within the exposure window examined (months 2–4 of gestation) [58].

**TABLE 1:** Study characteristics.

Author, date (ref)	Study design	Country (study period)	Disease status	Total sample size (pregnant people/neonates)	Primary 5‐HT_3_ antagonist exposure (number of exposed pregnancies)	Type of exposure reported (timing in pregnancy)	Comparator	Outcomes examined
Asker, 2005 [19]	Prospective cohort study	Sweden (1995–2002)	NVP including HG	665 572/676 198	Ondansetron (65)	Use in hospital records (Any trimester)	General population group	*Neonatal*: Congenital malformations, Birth characteristics
Einarson, 2004 [20]	Prospective cohort study	Canada; Australia (Unclear)	NVP including HG	528/528	Ondansetron (176)	Self‐reported use (First trimester, < 12 weeks gestation)	Other antiemetics (Diclectin ie Pyridoxine/Doxylamine, Metoclopramide, Phenothiazines and ginger); No antiemetic exposure	*Maternal*: Pregnancy complications *Neonatal*: Congenital malformations, Birth characteristics
Larrimer, 2014 [21]	Prospective cohort study	United States (Unclear)	NVP including HG	533/550	Ondansetron or Promethazine (78)	Self‐reported use (Any trimester)	No antiemetic exposure	*Neonatal*: Birth characteristics *Child*: Neurobehavioural outcomes
Sakran, 2021 [22]	Prospective cohort study	Israel (2010–2014)	NVP including HG	1083/1116	Ondansetron (195)	Self‐reported use (At least in first trimester)	Metoclopramide; Non‐teratogenic exposure	*Maternal*: Adverse effects, Pregnancy complications *Neonatal*: Congenital malformations, Birth characteristics
Ambrogi, 2025 [23]	Retrospective cohort study	United States (2020–2023)	NVP including HG	7023/7023	Ondansetron (1019)	Prescription dispensing (Unclear timing)	No Ondansetron exposure	*Maternal*: Pregnancy complications *Neonatal*: Birth characteristics
Berard, 2019 [24]	Retrospective cohort study	Canada (1998–2015)	NVP including HG	224 876/224, 876	Ondansetron (31)	Prescription dispensing (First trimester)	No antiemetic exposure	*Neonatal*: Congenital malformations
Chen, 2024 [25]	Retrospective cohort study	United States (2017–2023)	NVP including HG	15 727/15 727	Ondansetron (1064)	Self‐reported use (Unclear timing)	No Ondansetron exposure	*Neonatal*: Congenital malformations
Colvin, 2013 [26]	Retrospective cohort study	Australia (2002–2005)	NVP including HG	96 698/98 325	Ondansetron (251 pregnant people, 263 neonates)	Oral prescription dispensing (Any trimester)	No Ondansetron exposure	*Maternal*: Pregnancy complications *Neonatal*: Congenital malformations, Birth characteristics
Danielsson, 2014 [27]	Retrospective cohort study	Sweden (1998–2012)	NVP including HG	Unclear/1 501 434	Ondansetron (1349)	Self‐reported use & prescription dispensing (Early pregnancy)	Meclizine; General population group	*Neonatal*: Congenital malformations
Dormuth, 2021 [28]	Retrospective cohort study	Canada; United States; United Kingdom (2002–2016)	NVP including HG	406 627/456, 963	Ondansetron (150197)	Prescription dispensing (Any trimester)	Other antiemetics (Diclectin ie Pyridoxine/Doxylamine, Metoclopramide, Promethazine)	*Maternal*: Pregnancy complications *Neonatal*: Congenital malformations, Birth characteristics
Fejzo, 2016 [29]	Retrospective cohort study	United States (2007–2014)	HG only	1335/3396	Ondansetron (1070)	Self‐reported use (1–12 weeks gestation)	HG/No Ondansetron; No HG	*Maternal*: Pregnancy complications *Neonatal*: Congenital malformations, Birth characteristics
Huybrechts, 2018 [30]	Retrospective cohort study	United States (2000–2013)	NVP including HG	1 502 895/1 816 414	Ondansetron (88467)	Prescription dispensing (First 3 months of pregnancy)	No Ondansetron exposure	*Neonatal*: Congenital malformations
Huybrechts, 2020 [31]	Retrospective cohort study	United States (2000–2014)	NVP including HG	1 460 687/1 822 506	Ondansetron (23866)	IV prescription dispensing (First trimester)	No Ondansetron exposure	*Neonatal*: Congenital malformations
Lemon, 2021 [32]	Retrospective cohort study	United States (2006–2014)	NVP including HG	Unclear/33 677	Ondansetron (3733)	Oral & IV use in health records (First trimester, 0‐ < 14 weeks gestation)	No Ondansetron exposure	*Neonatal*: Congenital malformations
Masarwe, 2023 [33]	Retrospective cohort study	Israel (2012–2016)	NVP including HG	1548/1548	Ondansetron (774)	Oral prescription dispensing (Any trimester)	No Ondansetron exposure (pregnancies without NVP)	*Neonatal*: Congenital malformations, Birth characteristics
Ozdemirci, 2016 [34]	Retrospective cohort study	Türkiye (2006–2011)	HG only	185/185	Ondansetron (100)	IV use in health records (< 13 weeks gestation)	Chlorpromazine	*Maternal*: Pregnancy complications *Neonatal*: Congenital malformations, Birth characteristics
Pasternak, 2013 [35]	Retrospective cohort study	Denmark (2004–2011)	NVP including HG	9575/9575	Ondansetron (1970)	Prescription dispensing (Any trimester)	No 5‐HT_3_ antagonist exposure; Antihistamine exposure (Promethazine, Cyclizine, Meclizine)	*Maternal*: Pregnancy complications *Neonatal*: Congenital malformations, Birth characteristics
Suarez, 2021 [36]	Retrospective cohort study	United States (2014–2017)	NVP including HG	2620/Unclear	Ondansetron (1712)	Oral & IV prescription dispensing (2–20 weeks gestation)	Metoclopramide or Promethazine	*Maternal*: Pregnancy complications
Suarez, 2021 [37]	Retrospective cohort study	United States (2014–2017)	NVP including HG	2677/Unclear	Ondansetron (1742)	Oral & IV prescription dispensing (2–20 weeks gestation)	Metoclopramide or Promethazine	*Maternal*: Pregnancy complications *Neonatal*: Birth characteristics
Abas, 2014 [38]	RCT	Malaysia (2011–2012)	HG only	160/160	Ondansetron (80)	IV administered as part of study (≤ 16 weeks gestation)	Metoclopramide	*Maternal*: Adverse effects
Kashifard, 2013 [39]	RCT	Iran (2011–2012)	HG only	83/83	Ondansetron (49)	Oral administered as part of study (< 16 weeks gestation)	Metoclopramide	*Maternal*: Adverse effects
Oliveira, 2014 [40]	RCT	United States (2012–2013)	NVP including HG	30/Unclear	Ondansetron (13)	Oral administered as part of study (< 16 weeks gestation)	Pyridoxine/Doxylamine	*Maternal*: Adverse effects
Robson, 2021 [41]	RCT	United Kingdom (2018–2020)	NVP including HG	30/33	Ondansetron with dummy (8); Metoclopramide with Ondansetron (9)	Oral & IV administered as part of study (< 17 weeks gestation)	Metoclopramide with dummy; Double dummy	*Maternal*: Adverse effects *Neonatal*: Congenital malformations, Birth characteristics
Shahraki, 2016 [42]	RCT	Iran (2014–2015)	NVP including HG	188/188	Ondansetron (88)	Oral administered as part of study (4–16 weeks gestation)	Pyridoxine	*Neonatal*: Congenital malformations, Birth characteristics
Shayan, 2023 [43]	RCT	Iran (2017–2018)	NVP excluding HG	120/120	Ondansetron (60)	Oral administered as part of study (8–9 weeks gestation)	Jalinus Syrup (dried extracts of quince, ginger, vinegar, and honey)	*Maternal*: Adverse effects
Sullivan, 1996 [44]	RCT	United States (1993–1994)	HG only	30/30	Ondansetron (15)	IV administered as part of study (First & early second trimester)	Promethazine	*Maternal*: Adverse effects
Anderka, 2012 [45]	Case–control	United States (1997–2004)	NVP including HG	10 383/10 408	Any 5‐HT_3_ antagonist (53); Ondansetron (51)	Self‐reported use (First trimester)	No 5‐HT_3_ antagonist exposure; No Ondansetron exposure	*Neonatal*: Congenital malformations
Fejzo, 2015 [46]	Case–control	United States (2007–2011)	HG only	292/481	Ondansetron (213)	Self‐reported use (Unclear timing)	No Ondansetron exposure	*Child*: Neurobehavioural outcomes
Parker (BDS study), 2018 [47]	Case–control	United States (1997–2014)^a^	NVP including HG	5873/5873	Ondansetron (375)	Self‐reported use (First trimester)	Other prescription antiemetic drugs or IV fluids; No antiemetic exposure	*Neonatal*: Congenital malformations
Parker (NBDPS study), 2018 [47]	Case–control	United States (1997–2011)^a^	NVP including HG	6751/6751	Ondansetron (253)	Self‐reported use (First trimester)	Other prescription antiemetic drugs or IV fluids; No antiemetic exposure	*Neonatal*: Congenital malformations
Petersen, 2024 [48]	Case–control	United States (2005–2011)	NVP including HG	3658/Unclear	Ondansetron (252)	Self‐reported use (First trimester)	No 5‐HT_3_ antagonist exposure	*Neonatal*: Congenital malformations
Schrager, 2023 [49]	Case–control	United States (1997–2011)	NVP including HG	39 711/Unclear	Ondansetron (Unclear)	Self‐reported use (Unclear timing)	No antiemetic exposure	*Neonatal*: Congenital malformations
Werler, 2014 [50]	Case–control	United States (2007–2011)	Not specified. Assume NVP^b^	Unclear/2683	Ondansetron (125)	Self‐reported use (29–112 days after first day of last menstrual period)	No Ondansetron exposure	*Neonatal*: Congenital malformations
Zambelli‐Weiner, 2019 [51]	Case–control	United States (2000–2014)	NVP including HG	864 083/Unclear	Ondansetron (76330)	Oral & IV prescription dispensing (First trimester)	No antiemetic exposure	*Neonatal*: Congenital malformations
Ferreira, 2012 [52]	Case series	Canada (2002–2011)	HG only	14/17	Ondansetron (16)	Use in hospital records (6–24 weeks gestation)	None	*Neonatal*: Congenital malformations, Birth characteristics
Shapira, 2020 [53]	Retrospective cohort study	Israel (2013–2015)	NVP including HG	208/214	Granisetron (100)	IV use in hospital records (First and/or second trimester)	No Granisetron exposure	*Maternal*: Pregnancy complications *Neonatal*: Congenital malformations, Birth characteristics
Aleyasin, 2016 [54]	RCT	Iran (2011–2012)	HG only	32/Unclear	Granisetron (16)	Oral & IV administered as part of study (≤ 20 weeks gestation)	Promethazine	*Maternal*: Adverse effects
Kumari, 2025 [55]^c^	RCT	India (2023)	HG only	32/Unclear	Granisetron (16)	Oral & IV administered as part of study (≤ 20 weeks gestation)	Promethazine	*Maternal*: Adverse effects
Le, 2017 [56]	Case series	United States (2014–2015)	NVP excluding HG	15/15	Granisetron (3)	Transdermal use in health records (6–16 weeks gestation)	Ondansetron	*Maternal*: Adverse effects
Caritis, 2016 [57]	Pharmacokinetic study (single arm)	United States (Unclear)	NVP including HG	16/16	Granisetron (13)	Transdermal administered as part of study (12 − < 19 weeks gestation)	Granisetron IV	*Maternal*: Adverse effects

Abbreviations: HG, hyperemesis gravidarum; IV, intravenous; NVP, nausea and vomiting in pregnancy; RCT, randomised control trial.

^a^ Parker et al. was the only study which included two different data collection periods, extracted separately for this review.

^b^ Study considers ondansetron exposure in months 2–4 of gestation, with no details on indication/disease status. Assumed for NVP given exposure window.

^c^ Kumari (2025) and Aleyasin (2016) are identical in title, methodology, results and discussion reporting.

### 4.2 Ondansetron

#### 4.2.1 Maternal Health Outcomes

Maternal adverse effects (AEs) with gestational ondansetron use were investigated in seven studies. Four compared to metoclopramide [22, 38, 39, 41], and single studies compared to promethazine [44], pyridoxine and doxylamine [40], and a herbal extract, jalinus syrup (quince, ginger, vinegar, and honey) [43]. The occurrence of AEs was 26.5% in the largest study examined (195 exposed pregnancies) [22], with constipation, sedation, and drowsiness most frequently reported [22, 38, 40]. An RCT of 160 HG patients identified a reduced risk of drowsiness (12.5% vs. 30%; *p* = 0.01) or dry mouth (10% vs. 23.8%; *p* < 0.01) with ondansetron compared to metoclopramide [38]. Other small studies (< 120 pregnancies) identified no difference in AEs compared to pyridoxine/doxylamine [40], or did not report any maternal AEs with ondansetron exposure [39, 41, 43, 44].

Ten articles explored obstetric complications with gestational ondansetron use. Increased odds of a urinary tract infection (UTI) were identified among pregnant people prescribed ondansetron compared to those without (odds ratio [OR] 1.93; 95% confidence interval [CI] 1.53–2.42; *p* < 0.0001) [23]. Other studies reported on pregnancy loss (6 papers) [20, 22, 28, 29, 35, 36], and gestational hypertensive disease (4 papers) [23, 26, 34, 37]. Findings on pregnancy loss were mixed. Fejzo and colleagues (2016) observed a decreased risk of spontaneous abortion (weeks 1–12) in HG with 1070 exposed pregnancies compared to 771 untreated pregnancies (OR 0.09; 95% CI 0.06–0.13; *p* < 0.01) [29]. Another similar cohort study identified concordant findings [22]. The other four studies observed no difference in spontaneous abortion risk compared to alternative antiemetics [20, 28, 35, 36].

No increased risk of gestational hypertensive disease with ondansetron use was identified, even in the presence of a UTI [23]. A retrospective study of 2677 pregnancies revealed no difference in risk compared to metoclopramide or promethazine after adjustment for maternal demographics and medical history (adjusted OR [aOR] 0.87; 95% CI 0.68, 1.12) [37]. Similar null findings were identified for pregnancy‐induced hypertension [34], and preeclampsia alone [26], compared to chlorpromazine and no ondansetron exposure, respectively.

#### 4.2.2 Neonatal Health Outcomes

Congenital anomalies (CA) were the most frequently examined outcome (24 articles), namely cardiac anomalies (13 papers) and orofacial clefts (9 papers). Developing any CA overall was not influenced by ondansetron exposure during the first trimester [20, 24, 27, 32, 34, 35], or throughout pregnancy [22, 26, 28, 29].

Results regarding cardiac anomalies were conflicting. A recent cohort study of 1064 ondansetron‐exposed pregnancies identified the majority of congenital disorders reported were cardiac pathologies [25]. Three other articles of 81 412 neonates exposed in the first trimester identified an increased risk of cardiac malformations [27, 51], specifically ventricular septal defects (VSD) [32]. The relative risk of isolated VSD in one study lay between 1.7 (95% CI 0.1, 2.9) and 2.1 (95% CI 1.1, 4.0) across various methodological approaches, adjusting for maternal characteristics, comorbidities, and NVP history [32]. However, accounting for similar covariates in the two largest studies with over 1.8 million births each rendered their findings statistically non‐significant with first trimester oral or intravenous (IV) ondansetron use [30, 31]. Other observational studies of 5873 to 456 963 neonates also identified null findings if exposed in the first trimester or any time during pregnancy [28, 47, 49]. Among the smaller studies, no more than five exposed cases per article were identified [22, 29, 33, 52].

Similarly, there was conflicting evidence surrounding orofacial cleft development. Huybrechts et al. (2018) identified an elevated risk following first trimester ondansetron use among 88 467 neonates contrasted with 1 727 947 unexposed neonates (adjusted relative risk [RR] 1.25; 95% CI 1.04, 1.50) [30]. Like other studies, they also observed an increased risk of isolated cleft palate [30, 45, 47, 49]. However, investigating IV ondansetron use only, which likely improved the accuracy of exposure classification, rendered these findings statistically non‐significant [31]. A similar effect was reported by Zambelli‐Weiner and colleagues (2019) (aOR: 1.12; 95% CI 0.95, 1.33); however the substantially smaller sample may have limited the statistical power [51]. Different datasets within the same case–control study identified cleft palate risk was significantly elevated in the larger dataset (*n* = 6751; aOR 1.6; 95% CI 1.1, 2.3) but not the smaller (*n* = 5873; aOR 0.5; 95% CI 0.3, 1.0) [47]. Later analysis identified a tendency for differential participation among exposed cases that may account for this [48].

Other type‐specific CA were analyzed in seven studies. A significantly increased risk of urinary system anomalies was identified in two of three population‐based studies comparing ondansetron to non‐exposed pregnancies [26, 47, 51]. These were for renal agenesis (aOR 1.8; 95% CI 1.1, 3.0) [47], and collecting system anomalies (aOR 1.07; 95% CI 1.00, 1.16) [51]. The same studies presented similar increased risks of congenital diaphragmatic hernias (aOR 1.5; 95% CI 1.00, 2.4; aOR 1.40; 95% CI 1.05, 1.87) [47, 51]. Ondansetron exposure was not linked to craniosynostosis [49], neural tube defects [33, 45, 47], hypospadias [45, 47], or isolated club foot [50].

Perinatal outcomes were investigated in 15 studies [19, 20, 21, 22, 23, 26, 28, 29, 33, 34, 35, 37, 41, 42, 52], the most common being stillbirth (7 papers) and preterm birth (8 papers). Neonatal birthweight and/or gestational age were also examined [20, 21, 22, 26, 33, 34, 35, 42]. Three studies reported low frequencies of perinatal outcomes (< 5 exposed cases) or lacked comparative analysis [19, 41, 52].

Rates of stillbirth did not differ between 3303 ondansetron‐exposed and 106 433 unexposed neonates across three retrospective cohort studies [26, 29, 35]. This was consistent when comparing ondansetron to chlorpromazine, or combinations of pyridoxine/doxylamine, metoclopramide, or promethazine [20, 28, 34, 37]. Preterm birth (< 37 weeks gestation) was also not associated with gestational ondansetron exposure in most studies [21, 22, 33, 34, 37]. Any increased risks were attenuated by analyzing live births only [29, 35]. Ondansetron was associated with greater odds of preterm birth in the presence of a UTI history (OR 2.59; 95% CI 1.47, 4.58; *p* = 0.0021) although authors note the existing association between gestational UTIs and preterm birth [23].

Frequency of low birth weight (< 2500 g) did not differ between ondansetron‐exposed and unexposed groups among the 108 085 neonates studied [26, 34, 35]; neither did the proportion of small for gestational age neonates in the 14 350 studied [21, 33, 35, 37]. Of the six studies measuring neonatal birthweight, five demonstrated no significant difference following ondansetron use [20, 22, 33, 34, 42]. Gestational ages at birth also appeared unaffected [20, 22, 33, 42]. However, a prospective study of 550 live births observed a significantly lower birth weight (3200 g vs. 3310 g; *p* = 0.023) and younger gestational age (38.6 weeks vs. 38.9 weeks; *p* = 0.016) among ondansetron‐ or promethazine‐exposed neonates compared to those without antiemetics [21].

#### 4.2.3 Child Health Outcomes

Two studies investigated the long‐term health of children exposed to ondansetron in utero [21, 46]. At 18 months of age, no differences were observed in neurobehavioural outcomes such as emotional reactivity and attention between infants exposed to ondansetron or promethazine and those without antiemetics [21]. Ondansetron use among 312 HG‐affected pregnancies also revealed no added risk of neurodevelopmental delay in later childhood (average age of follow up = 8 years old) [46].

### 4.3 Granisetron

#### 4.3.1 Maternal Health Outcomes

Five studies examined granisetron safety in 164 exposed pregnant people [53, 54, 55, 56, 57]. Only the pharmacokinetics study of 28 participants receiving either IV or transdermal granisetron identified maternal AEs [57], with constipation and headaches most frequently reported. Other side effects were localized (e.g., rashes) with the transdermal patch [57]. The only study expanding on maternal health outcomes identified a significantly reduced rate of spontaneous abortion in 100 granisetron‐exposed pregnancies compared to 108 unexposed controls (0% vs. 5.5%; *p* = 0.03) [53]. The limited sample size may account for the low rates of spontaneous abortion below the expected background rate (~15%) [59].

#### 4.3.2 Neonatal Health Outcomes

In the only study examining neonatal outcomes following gestational granisetron use [53], three instances of major malformations were identified: a diaphragmatic hernia, and two cardiac septal defects. The calculated occurrence of CA events (*n* = 3/108, 2.8%) did not significantly differ from the unexposed neonates (*n* = 3/106, 2.8%). Neither group exhibited cases of perinatal mortality. At birth, neonatal anthropometric characteristics and gestational age were not significantly different between groups.

## 5 Discussion

The review of published evidence to date suggests ondansetron is likely associated with low risks to pregnant people and their neonates in the treatment of NVP. However, inconsistencies exist for prenatal ondansetron exposure and certain type‐specific anomalies. Research into the safety of granisetron or other 5‐HT_3_ antagonists in pregnancy appears extremely limited.

Maternal AEs with ondansetron use were typically mild and transient, similar to other antiemetics for NVP [60]. Serious rare side effects may include seizures or long QT syndrome [60], often occurring with a single high‐dose ondansetron administration above 8 mg [10]. Studies in this review were likely limited in their ability to detect rare, severe AEs by their small sample sizes and use of oral or 4 mg IV formulations. A case report of serotonin syndrome following NVP treatment with ondansetron has been previously described [61], however other serotonergic medications (e.g., sumatriptan) may have contributed to this outcome.

The evidence suggests ondansetron may protect against spontaneous abortion, although this was not consistent across all studies. NVP itself is associated with a reduced risk of pregnancy loss [1, 62], therefore studies that inadequately control for NVP may introduce confounding by indication if the results reflect NVP symptoms rather than the action of ondansetron itself. Articles demonstrating no increased risk of spontaneous abortion with ondansetron utilised larger sample sizes and compared alternative antiemetics to better account for disease status [20, 28, 35, 36].

Ondansetron's teratogenic profile is conflicting in both this review and others [11, 12]. The few articles identifying positive signals for cardiac anomalies were likely compromised by selective adjustment for covariates, ascertainment bias in diagnosing VSD, or conflicts of interest in the case of Zambelli‐Weiner et al. (2019) [63]. Evidence from larger epidemiological studies suggested no increased risk of any cardiac anomalies with ondansetron use [28, 30, 47, 49]. Regarding orofacial clefts, the marginal changes in relative risk may be outweighed by the benefits of treatment at a population level. For example, Huybrechts et al. (2018) identified the statistically‐significant association equates to 3 additional cases of orofacial clefts per 10 000 treated pregnancies [30]. The weak positive signal for urogenital and diaphragmatic anomalies may warrant further investigation, given the small sample sizes present [47, 51]. Overall, the frequency of CA in the included articles fell well below the expected background rate (~6%) [64]. This casts doubt on the true teratogenic effects of ondansetron in excess of baseline risk.

Ondansetron did not appear to adversely affect other neonatal outcomes, such as stillbirth or preterm birth, which is consistent with recent meta‐analyses [12]. These late pregnancy outcomes are largely impacted by third trimester exposures, when absolute fetal growth is a priority [13], by which time NVP symptoms and associated treatment have likely ceased. In severe NVP cases requiring treatment throughout pregnancy, these statistically significant differences may lack clinical significance in practice. For example, differences in birthweight by 110 g between exposed and control neonates were not clinically meaningful [21].

Granisetron has demonstrated greater antiemetic effects over ondansetron in non‐pregnant patients [65, 66]. However, only small, single studies were identified for maternal and neonatal health outcomes in pregnancy, precluding strong conclusions from being drawn. Therefore, the current lack of safety evidence suggests granisetron should be avoided for NVP. Considering other 5‐HT_3_ antagonists used in non‐pregnant populations [67, 68], there was no evidence to support their use for NVP.

Despite the growing body of evidence surrounding 5‐HT_3_ antagonist use in pregnancy, its clinical utility is limited by the extreme heterogeneity between studies. The definitions of NVP and HG vary widely across self‐report methods and clinical diagnoses, hence objective and validated assessment for disease severity (e.g., PUQE‐24) [69] should be considered to minimise confounding. Furthermore, many studies were likely underpowered to assess rare risks, which may bias results towards the null. Both cardiac and orofacial anomalies have an estimated population prevalence of 82/10000 [70] and 14/10000 [71] births respectively; of which only the two largest studies of over 1.8 million pregnancies identified approximations similar to these [30, 31]. Therefore, additional population‐based studies with larger sample sizes would be of value. Expanding the current literature may involve examining long‐term child health outcomes or exploring consumer perspectives through qualitative approaches.

National guidelines from the UK [72], USA [4], Canada [73], and Australia [74], are synonymous in not recommending ondansetron as first‐line therapy for NVP citing insufficient safety data in pregnancy is present [4, 72]. The UK and Australian recommendations also note the insufficient evidence on granisetron [72, 74]. Considering the evidence explored, ondansetron appears to pose minimal risk to maternal health and neonatal outcomes (excluding CA). Therefore, ondansetron use should not be discouraged if first‐line management fails, given the detrimental effects of untreated NVP of any severity [1]. With respect to CA, pregnant people resorting to ondansetron can be reassured that any risks of type‐specific CA are minimal. Ultimately, the combined perspectives of the pregnant person and their healthcare team are essential in balancing the risks of ondansetron use against that of poorly managed NVP.

### 5.1 Strengths and Limitations

While existing reviews often focus exclusively on fetal outcomes [11, 12], this paper deliberately synthesizes maternal and obstetric outcomes to address this gap. This review is also novel in examining the entire 5‐HT3 antagonist medication class available, as opposed to ondansetron alone. Observing a broad range of comparator groups including prescription medications, over‐the‐counter formulations, and an herbal therapy also provided a comprehensive frame of reference across many clinical settings.

Limitations include the restricted selection criteria, which excluded grey literature and non‐English literature. Furthermore, this review does not formally account for the heterogeneity between studies, both in design and risk estimate reporting, by way of a meta‐analytic approach. This was an informed decision given the substantial variability of evidence across outcomes such as miscarriage (I^2^ = 92%), preterm birth (I^2^ = 85%) and cardiac defects (I^2^ = 53%) in previous meta‐analyses [12]. Overlapping datasets used across some studies also suggest the pregnancies included in this review are not all unique. These factors may have contributed towards the varied effects measures reported.

### 5.2 Conclusions

Ultimately, literature surrounding 5‐HT_3_ antagonist medications for NVP is dominated by ondansetron, which appears to pose a low risk of harm to maternal health, with a marginally increased risk of certain type‐specific anomalies which is yet to be fully elucidated. Therefore, ondansetron should still be considered a viable second‐line treatment option for NVP, if discussed alongside its potential risks. The paucity of literature on granisetron provides limited knowledge of its safety profile in pregnancy, hindering its widespread use at present. As emerging research increasingly relies on large population‐based data sources, small but statistically significant risk estimates are likely to emerge. Appropriately contextualising these results will be essential in interpreting future research and providing safe, effective clinical care for pregnant people.

## Author Contributions

E.K. and S.M. were responsible for the conception and planning of the study. All authors contributed to the acquisition of data. Data interpretation and preparation of a first draft manuscript was completed by S.M. under the supervision of E.K. All authors were involved in editing of the manuscript for publishing.

## Funding

This work was supported by the National Health and Medical Research Council (NHMRC) Centre of Research Excellence in Medicines Intelligence (ID:1196900). Erin Kelty is supported by the NHMRC Fellowship (ID:1172978).

## Ethics Statement

The authors have nothing to report.

## Conflicts of Interest

The authors declare no conflicts of interest.

## Supporting information


**Table S1:** Search strategy adapted to specific inputs of each interface (OVID or EBSCO). Publication date limited from 20/03/2025 to current for updated search. Updated search date: 07/05/2025.

## Data Availability

Data sharing not applicable to this article as no datasets were generated or analysed during the current study.
