# Supplementary material for: A Scoping Review of 5‐HT3 Antagonist Antiemetic Medication Safety in the Management of Nausea and Vomiting of Pregnancy
Source: Pharmacoepidemiol Drug Saf. 2026 Aug 13;35(9):e70449. doi: 10.1002/pds.70449 (PMC13470839; doi:10.1002/pds.70449)
Supplement: Supplementary file 1 [file PDS-35-e70449-s001.docx]

**Supplementary material**

**A scoping review of 5-HT3 antagonist antiemetic medication safety in the management of nausea and vomiting of pregnancy**

**In: *Pharmacoepidemiology and Drug Safety***

Shannon Morgan,^a^ Ebony Quintrell,^b^ Danielle J. Russell,^b^ Amina Rhaman,^b^ Aster Gebremedhin,^c^ Caitlin S. Wyrwoll,^c^ & Erin Kelty.^b^

*^a^ Medical School, The University of Western Australia, Perth, Australia;*

*^b^ School of Population & Global Health, The University of Western Australia, Perth, Australia;*

*^c^ School of Human Sciences, The University of Western Australia, Perth, Australia.*

*Corresponding author:*	Dr Erin Kelty ([erin.kelty@uwa.edu.au](mailto:erin.kelty@uwa.edu.au))

***Table S1*** Search strategy adapted to specific inputs of each interface (OVID or EBSCO). Publication date limited from 20/03/2025 to current for updated search. Updated search date: 07/05/2025

| **#** | **Search terms** |
| --- | --- |
| Embase, Ovid Medline, Global Health | |
| 1 | (5-HT3 antagonists or Serotonin 5-HT3 Receptor Antagonists or ondansetron or Ondansetron or Zofran or Zotren or Zuplenz or setron or tropisetron or Tropisetron or Novartis or Nabovan or granisetron or Kytril or Granisetron or dolasetron or Anzemet or palonosetron or Palonosetron or Aloxi or ramosetron or Irribow).ti,ab. |
| 2 | exp Pregnancy/ or exp Pregnancy Complications/ or exp Obstetrics/ or exp Labor Pain/ or (parturition or ante natal* or antenatal* or pre natal* or prenatal* or puerper* or postnatal* or postpartum or post partum or post natal* or peripartum or peri partum or prepregnancy or pre pregnancy or preconception* or pre conception* or periconception* or peri conception* or ((preterm or premature) and (labor or labour)) or eclamp* or preeclamp* or pre eclamp* or amniocentes* or chorion* vill* or cesarean or caesarean or cesarian or caesarian or cesarien or caesarien or newborn* or new born* or tocoly* or fetal or foetal or fetus or foetus or miscarriage* or pregnancy or pregnancies or pregnant).ti,ab. |
| 3 | #1 AND #2 |
| Cumulated Index to Nursing and Allied Health Literature (CINAHL) | |
| 1 | MH +(5-HT3 antagonists or Serotonin 5-HT3 Receptor Antagonists) |
| 2 | ondansetron or Ondansetron or Zofran or Zotren or Zuplenz or setron or tropisetron or Tropisetron or Novartis or Nabovan or granisetron or Kytril or Granisetron or dolasetron or Anzemet or palonosetron or Palonosetron or Aloxi or ramosetron or Irribow |
| 3 | Pregnancy or Pregnancy Complications or Obstetrics or Labor Pain/ or (parturition or ante natal* or antenatal* or pre natal* or prenatal* or puerper* or postnatal* or postpartum or post partum or post natal* or peripartum or peri partum or prepregnancy or pre pregnancy or preconception* or pre conception* or periconception* or peri conception* or ((preterm or premature) and (labor or labour)) or eclamp* or preeclamp* or pre eclamp* or amniocentes* or chorion* vill* or cesarean or caesarean or cesarian or caesarian or cesarien or caesarien or newborn* or new born* or tocoly* or fetal or foetal or fetus or foetus or miscarriage* or pregnancy or pregnancies or pregnant |
| 4 | #1 OR #2 AND #3 |
